# The Postprandial Glycaemic and Hormonal Responses Following the Ingestion of a Novel, Ready-to-Drink Shot Containing a Low Dose of Whey Protein in Centrally Obese and Lean Adult Males: A Randomised Controlled Trial

**DOI:** 10.3389/fendo.2021.696977

**Published:** 2021-06-18

**Authors:** Kieran Smith, Guy S. Taylor, Dean M. Allerton, Lise Hoej Brunsgaard, Kelly A. Bowden Davies, Emma J. Stevenson, Daniel J. West

**Affiliations:** ^1^ Population Health Sciences Institute, Newcastle University, Newcastle upon Tyne, United Kingdom; ^2^ Health and Performance Nutrition, Arla Foods Ingredients Group P/S, Viby J., Denmark; ^3^ School of Sport and Exercise Sciences, Manchester Metropolitan University, Manchester, United Kingdom

**Keywords:** gastric emptying, GLP-1 - glucagon-like peptide-1, whey protein, metabolic syndrome, GIP - glucose-dependent insulinotropic peptide, central obesity, incretin peptides, postprandial glycaemia

## Abstract

**Purpose:**

Elevated postprandial glycaemia [PPG] increases the risk of cardiometabolic complications in insulin-resistant, centrally obese individuals. Therefore, strategies that improve PPG are of importance for this population. Consuming large doses of whey protein [WP] before meals reduces PPG by delaying gastric emptying and stimulating the secretion of the incretin peptides, glucose-dependent insulinotropic polypeptide [GIP] and glucagon-like peptide 1 [GLP-1]. It is unclear if these effects are observed after smaller amounts of WP and what impact central adiposity has on these gastrointestinal processes.

**Methods:**

In a randomised-crossover design, 12 lean and 12 centrally obese adult males performed two 240 min mixed-meal tests, ~5–10 d apart. After an overnight fast, participants consumed a novel, ready-to-drink WP shot (15 g) or volume-matched water (100 ml; PLA) 10 min before a mixed-nutrient meal. Gastric emptying was estimated by oral acetaminophen absorbance. Interval blood samples were collected to measure glucose, insulin, GIP, GLP-1, and acetaminophen.

**Results:**

WP reduced PPG area under the curve [AUC_0–60_] by 13 and 18.2% in the centrally obese and lean cohorts, respectively (both p <0.001). In both groups, the reduction in PPG was accompanied by a two-three-fold increase in GLP-1 and delayed gastric emptying. Despite similar GLP-1 responses during PLA, GLP-1 secretion during the WP trial was ~27% lower in centrally obese individuals compared to lean (p = 0.001). In lean participants, WP increased the GLP-1_ACTIVE/TOTAL_ ratio comparative to PLA (p = 0.004), indicative of reduced GLP-1 degradation. Conversely, no treatment effects for GLP-1_ACTIVE/TOTAL_ were seen in obese subjects.

**Conclusion:**

Pre-meal ingestion of a novel, ready-to-drink WP shot containing just 15 g of dietary protein reduced PPG in lean and centrally obese males. However, an attenuated GLP-1 response to mealtime WP and increased incretin degradation might impact the efficacy of nutritional strategies utilising the actions of GLP-1 to regulate PPG in centrally obese populations. Whether these defects are caused by an individual’s insulin resistance, their obese state, or other obesity-related ailments needs further investigation.

**Clinical Trial Registration:**

ISRCTN.com, identifier [ISRCTN95281775]. https://www.isrctn.com/.

## Introduction

The prevalence of obesity is increasing globally, which poses a significant challenge to health care systems. Obesity is a well-defined risk factor for the development of cardio-metabolic complications such as cardiovascular disease [CVD] and type 2 diabetes [T2D] (1, 2). In fact, obesity increases the risk of T2D six-fold, irrespective of genetic risk (3). Yet not all obese individuals develop T2D or display dysglycaemia. Instead, fat distribution is a critical determinant of insulin sensitivity (4), specifically when fat is stored around visceral areas (5–7), which is associated with hepatic insulin resistance, dyslipidaemia and impairments in insulin-mediated peripheral glucose disposal (8).

In the years that precede the transition from normal glucose tolerance [NGT] to T2D, a progressive decline in both insulin’s action and its secretion occurs augmenting a steady decline in glucose tolerance (9). For individuals who develop T2D, evidence suggests that it is the deterioration in postprandial glycaemia [PPG], rather than fasting glycaemia, that precedes the decline to overt dysglycaemia (10). Notably, PPG excursions have been shown to independently predict future cardiovascular morbidity and mortality even when fasting blood glucose concentrations or HbA_1c_ are normalised (11–13). Therefore, approaches to minimising PPG are of importance for both patient and non-patient populations.

Several lines of work demonstrate that mealtime whey protein [WP] supplementation may serve as a simple, non-pharmaceutical approach to improve PPG control [as reviewed (14)]. WP is rich in branched-chain amino acids and bioactive peptides [potentially β-lactoglobulin and α-lactalbumin (15)] that upon digestion are rapidly absorbed into circulation. These constituents are potent insulin secretagogues (16) that also augment the incretin effect through the release of incretin peptides, glucose-dependent insulinotropic polypeptide [GIP] and glucagon-like peptide 1 [GLP-1] (15, 17), and delay the rate of gastric emptying (18). However, where a wealth of evidence demonstrates that pre-meal WP supplementation attenuates glycaemic excursions in obese individuals with T2D (19–23), studies investigating this treatment in obese subjects without overt dysglycaemia are scarce (24). This is surprising given such populations are likely exposed to periods of postprandial hyperglycaemia (25) and are at an increased risk of CVD and T2D (1, 2). There is also evidence to suggest that obese individuals have reduced gastrointestinal sensitivity to dietary nutrients (26–28), which may compromise the effectiveness of dietary preloads to regulate PPG. However, the gastrointestinal responses to mealtime WP in obese individuals remain poorly characterised.

Clinically meaningful improvements in metabolic health require chronic changes in PPG and other cardio-metabolic markers (29). It is, therefore, imperative to consider the long-term application of an available treatment and its sustainability to improve metabolic health. To this end, there are several methodological limitations associated with mealtime WP supplementation that have not been addressed in previous studies (14). For instance, it is common practice to present WP preloads as unpalatable, dry-mix powders that require dilution and mixing with flavouring immediately prior to their consumption (20–22, 24). However, there is a general unwillingness to consume dry-mix protein supplements in the presence of others (30) with taste and convenience also determining eating behaviours (31). These observations suggest that current preloading strategies are unlikely to be applicable beyond the research setting.

Accordingly, the purpose of this study was twofold. Firstly, given the associations between central adiposity and the development of T2D (1, 2, 11), we examined the glucose-lowering potential of a low volume, ready-to-drink WP shot innovated specifically as a non-pharmaceutical agent for PPG control in centrally obese adult males. Secondly, we assessed the practical application of our novel WP shot, and examined the hormonal and gastrointestinal responses to its ingestion in obese and healthy states.

## Methods

### Participants

Adult male volunteers aged between 18 and 65 y were recruited from the North-East of England. All participants regularly consumed breakfast, adhered to a standard sleep–wake cycle, were non-smokers, free from metabolic disease and reported no known food or dietary intolerances. Based upon the World Health Organisation’s threshold associated with the greatest risk of metabolic complications within Europid males, central adiposity was determined if an individual presented with a waist circumference of ≥102 cm or had a BMI ≥30 kg/m^2^ (32). Further, physically active individuals with a BMI ranging between 18.9 and 24.9 kg/m^2^ or a waist circumference of ≤94 cm were recruited as healthy controls (32). Ethical approval granted by the Ethics Committee of the Faculty of Medical Sciences, Newcastle University, and written informed consent was obtained from all participants at least 72 h prior to study enrolment. The trial was registered at ISRCTN.com (ISRCTN95281775).

### Study Design

Participants were entered into a randomised-control, counterbalanced, crossover design involving two laboratory-based feeding trials. Participants consumed a WP or control [PLA] beverage 10 min before a standardised breakfast and provided venous blood samples over a 240 min postprandial period. Experimental visits were separated by 5–10 d.

### Pre-Laboratory Control

Strenuous bouts of physical activity and alcohol were to be avoided 24 and 48 h prior to each experimental visit, respectively. Participants were instructed to avoid taking analgesics containing paracetamol the day preceding entry to the laboratory. Habitual dietary intake was recorded 24 h prior to participant’s first mixed-meal tolerance test and was replicated prior to the subsequent trial. A standardised evening meal (897 kcal [3,753 kJ]: 58% carbohydrates, 23% fat, 19% protein) was provided to be consumed the evening prior (~1,900–2,100 h) to entering the laboratory.

### Mixed Meal Tolerance Test

Participants reported to the laboratory following a ~12 h overnight fast (0800 h ± 1 h). Once rested, an intravenous cannula (B Braun, Germany) was introduced into an antecubital vein for repeated blood sampling and a fasting sample collected (t = −15 min). Ten minutes prior to a mixed-nutrient breakfast (t = −10 min), participants randomly consumed a WP shot (15.6 g protein; Lacprodan^®^ DI-6820, Arla Foods Ingredients Group P/S, Denmark) or volume-matched water [100 ml; PLA]. The mixed-nutrient breakfast consisted of 60 g ready-to-eat cereal (Cheerio’s, Nestle, UK) and 250 ml whole milk, providing each participant with 387 kcal (1,619 kJ) of energy derived from 58% carbohydrates, 27% fat, and 15% protein. The breakfast meal was initiated at t = −0 min and was to be consumed within 15 min to standardise any effects of eating rate on postprandial hormonal responses. Postprandial venous blood samples, and subjective appetite parameters *via* completion of a paper-based visual analogue scale [VAS], were collected periodically over a 240 min postprandial period (t = 0–240 min).

For the assessment of gastric emptying, participants were provided with 100 ml water and 1,500 mg of paracetamol (Bristol Laboratories Ltd, United Kingdom) that was consumed orally at the commencement of breakfast. Orally administered acetaminophen (paracetamol) is poorly absorbed by the stomach but is rapidly absorbed within the small intestine; thus, gastric emptying is the rate-limiting step for the appearance of acetaminophen within blood (33). The time to reach maximum acetaminophen concentrations occurs ~30–60 min post-ingestion; therefore, area under the curve during the first 60 min [AUC_0–60_] is regarded as a marker of the velocity of gastric emptying rates.

Water consumption during the postprandial period was limited to 250 ml per trial. To control for postural changes in plasma volume, participants remained seated throughout the testing period. Pre-arranged transportation to the facilities was provided to reduce pre-trial physical exertion.

#### Whey Protein Beverage

Participants were provided with a protein-rich, low volume, ready-to-drink WP shot of low viscosity liquid (15.6 g of WP in 100 ml). The pre-meal shot utilised a hydrolysed WP ingredient (Lacprodan^®^ DI-6820, Arla Food Ingredients Group P/S) to produce a palatable, ready-to-drink beverage with a shelf life of 6 months that was stable at both room temperature and chilled environmental conditions. The design of this pre-meal shot was an academic-industry collaboration that incorporated the maximum dose of WP available to be present in minimal liquid volume, whilst taking into consideration the energy associated (100 kcal [418 kJ]), taste and mouthfeel of the WP product, and consumer convenience and preference. Each participant was provided with a “cocoa–cappuccino” flavoured WP shot and assessed the preload’s palatability *via* completion of a VAS. Please see supplementary data for detailed product development information (**Supplementary Material 1**).

#### Blood Collection and Analytical Procedures

Venous whole blood samples were collected into EDTA and serum vacutainers (Becton Dickinson, USA), and FC Mix vacuettes (Greiner Bio-One, Austria). EDTA vacutainers were pre-treated with aprotinin (#A6279, Sigma Aldrich, USA) and a DPP-IV inhibitor (#DPP4-010, Merck Millipore, USA) for the preservation of active GLP-1 [GLP-1_ACTIVE_] and kept on ice. EDTA and serum vacutainers were centrifuged at 3,000 rpm at 4°C for 10 min with the corresponding plasma and serum supernatant transferred into aliquots and stored at −80°C until subsequent analysis.

Serum insulin concentrations were quantified using an ELISA with <0.01% cross-reactivity with C-peptide or Proinsulin, and an assay sensitivity of 6 pmol/L (intra-assay, 3.8%; inter-assay, 9.7%; #10-1113-01, Mercodia AB, Sweden).

Plasma GLP-1 were quantified using a C-terminal targeting ELISA that measured the amidated forms of GLP-1, with a sensitivity of 1.5 pmol/L (intra-assay, 5%; inter-assay, 6.4%; #EZGLP1T-36K, Merck Millipore). This measurement reflects both the total amount of GLP-1 secreted (herein referred to as “GLP-1”) but also the peptide’s biological actions given GLP-1 activates afferent sensory neurons in the gastrointestinal tract before being degraded in the capillaries of the gut (34). GLP-1_ACTIVE_ was measured using an ELISA employing N-terminal and side-viewing antibodies that measured GLP-1_[7–36]_ NH_2_ with no cross-reactivity with GLP-1_[7–37]_, the primary GLP-1 metabolite (GLP-1_[9–36]_ NH_2_/_[9–37]_), or N-terminally extended forms (intra-assay, 10.3%; inter-assay, 14.1%; #80-GP1A-CH01, Alpco, USA). Plasma GIP were analysed using a C-terminal targeting ELISA that measured both the GIP_[1–42]_ and GIP_[3–42]_ moiety with an assay sensitivity of 1 pmol/L (intra-assay, 3.3%; inter-assay, 11.3%; #EZHGIP-54K, Merck Millipore).

HDL cholesterol, total cholesterol and fasting triglyceride concentrations were determined using plasma on an automated benchtop analyser (Reflotron Plus, Roche Diagnostics, USA) with an intra-analyte variation of <2.6, <5.6 and <8.1%, respectively. Plasma glucose concentrations were calculated from venous whole blood collected in FC Mix vacuettes (intra-assay, 5.7%; Biosen C_Line, EKF Diagnostics, UK). Serum acetaminophen concentrations were analysed using a clinical analyser (Roche Cobas, Roche Diagnostics GmBH, Germany) with a detection limit of 7.94mol/L (#05841208, Roche Diagnostics GmBH). Acetaminophen measures were conducted by the Blood Science unit of the Royal Victoria Infirmary (Newcastle upon Tyne Hospitals, UK). Where possible, samples from each participant were analysed on the same assay plate.

### Calculations

Total and incremental areas under the curve [AUC_n_ and iAUC_n_, respectively] were calculated using the trapezoidal rule (35) and divided by the duration of the observational period of interest (i.e., 60 or 240 min) to provide time-averaged values. The iAUC included all area below the concentration curve, ignoring values below baseline concentrations (35). HOMA-IR, which best reflects hepatic insulin resistance, was calculated using basal glucose and insulin concentrations from both mixed meal tolerance tests (36). Subjective appetite parameters (fullness, hunger, satisfaction, and prospective food intake) from the paper-based VAS were combined to produce a combined-appetite score (37). Missing sample points (1 time point from one lean and one centrally obese participant during the PLA and WP trial, respectively) were imputed by linear interpolation.

### Sample Size Calculation

A sample size calculation was performed based on prior data collected from our laboratory using PPG AUC as the primary outcome (19, 24). To detect a statistical difference in PPG AUC, 12 participants were required in each group to test the null hypothesis that the population means of both groups are equal with probability of 0.8 and an associated type 1 error of 0.05.

### Data Analysis

All data was assessed for normal distribution by a Shapiro–Wilks test and investigation of boxplots for outliers. Non-parametric data was log transformed and re-assessed for distribution. Where transformation failed, data were assessed non-parametrically. Between-group baseline variables were analysed by an independent samples t test or a Mann–Whitney U test. A mixed-model ANOVA with repeated measures with two within-group (*time* and *treatment* [i.e., WP or PLA]) and one between-group factors (*condition* [i.e., lean or centrally obese]) was performed to assess time-course changes in acetaminophen, glucose, insulin, the incretin peptides, and subjective appetite. AUC and iAUC variables (acetaminophen, hormonal, and subjective appetite) were analysed by a two-way mixed ANOVA with *treatment* and *condition* as factors. Post hoc analysis, adjusted for multiple comparisons by Bonferroni correction, were performed if ANOVAs revealed any significant interaction effects. Postprandial insulin and GLP-1_ACTIVE_ concentrations were analysed by a two-way Friedman’s rank test with pairwise comparison to locate within-group treatment effects, and by a Kruskal–Wallis H test to examine between-group differences during the two trials. The Pearson product-moment correlation (*r*) and the Spearman’s rank order correlation (r_s_) were used to explore associations between variables displaying normal or non-normal distribution, respectively. Inferential statistics was conducted using the software package IBM SPSS Statistics (Version 26; IBM Corp., USA) and presented graphically using GraphPad Prism (GraphPad software version 9.0, USA). Significance was set at alpha p <0.05. All data is presented as means ± standard deviation [SD] unless stated otherwise.

## Results

### Participant Characteristics

The CONSORT [Consolidated Standards of Reporting Trials] flow diagram is shown in **Supplementary Material 2**. Twenty-five adult males (n = 12 centrally obese; n = 13 lean) were recruited and completed this investigation. One lean individual was highlighted as insulin-resistant (HOMA-IR 3.5) compared to the rest of the lean cohort and was subsequently removed from analysis. Therefore, data is analysed on 24 participants (n = 12 centrally obese; n = 12 lean). Participant characteristics are presented in **Table 1**.

**Table 1:** Cohort characteristics.

	Lean (n = 12)	Centrally Obese (n = 12)
	Characteristics	
**Age (years)**	35.8 ± 10.6	34.8 ± 7.4
**Stature (cm)**	177.4 ± 5.3	181.5 ± 5.3
**Body mass (kg)**	74.7 ± 7.2	111.1 ± 12.1*
**Body mass index (kg/m^2^)**	23.7 ± 1.8	33.7 ± 2.4*
**Waist circumference (cm)**	80.4 ± 6.4	110.2 ± 10.1*
**Hip circumference (cm)**	95.4 ± 4.6	114.2 ± 10.8*
**Waist-to-hip ratio**	0.84 ± 0.04	0.97 ± 0.07*
**SBP (mmHg)**	118 ± 9.3	134.4 ± 8.6*
**DBP (mmHg)**	70.7 ± 6.8	81.3 ± 7.9*
	**Fasting Metabolic Variables**	
**Fasting glucose (mmol/L)**	4.40 ± 0.26	4.56 ± 0.4
**Fasting insulin (pmol/L)**	51.58 ± 17.14	101.94 ± 45.41*
**HOMA-IR**	1.68 ± 0.56	3.40 ± 1.40*
**GLP-1 (pmol/L)**	17.29 ± 4.63	25.78 ± 9.24*
**GLP-1**_ACTIVE_ **(pmol/L)**	0.79 ± 0.40	0.78 ± 0.53
**GLP-1**_ACTIVE/TOTAL_ **(pmol/L)**	0.05 ± 0.03	0.03 ± 0.02*
**GIP (pmol/L)**	11.01 ± 5.60	11.42 ± 4.67
**TC (mmol/L)**	4.22 ± 0.85	3.85 ± 0.66
**HDL (mmol/L)**	1.21 ± 0.27	0.95 ± 0.10*
**Tg (mmol/L)**	1.15 ± 0.24	1.60 ± 0.37*

DBP, diastolic blood pressure; GIP, glucose-dependent insulinotropic polypeptide; GLP-1, glucagon-like peptide 1; HDL, high-density lipoprotein; HOMA-IR, homeostatic model assessment—insulin resistance; SBP, systolic blood pressure; TC, total cholesterol; Tg, triglycerides.

Data presented are mean averages from duplicate measurements taken during both trials. Lipid markers were measured from each participant’s first trial.

All data is presented as mean ± SD.

*Denotes a statistical significance between groups as determined by an independent samples t test or a Mann–Whitney U Test (p <0.05).

All subjects displayed normoglycemic fasting blood glucose concentrations; however, fasting insulin concentrations were markedly greater in the centrally obese cohort. Subsequently, HOMA-IR scores were greater in centrally obese participants. Fasting GLP-1 concentrations were also greater in this group. However, relative to total GLP-1, centrally obese individuals had lower GLP-1_ACTIVE_ concentrations, indicative of increased dipeptidyl-peptidase IV [DPP-IV] activity in the basal state. When assessing participant characteristics individually, five subjects from the centrally obese cohort displayed three or more characteristics associated with the Metabolic Syndrome (32). One centrally obese subject had a family history of diabetes.

### Postprandial Responses

#### Plasma Glucose Concentrations

**Figure 1:**
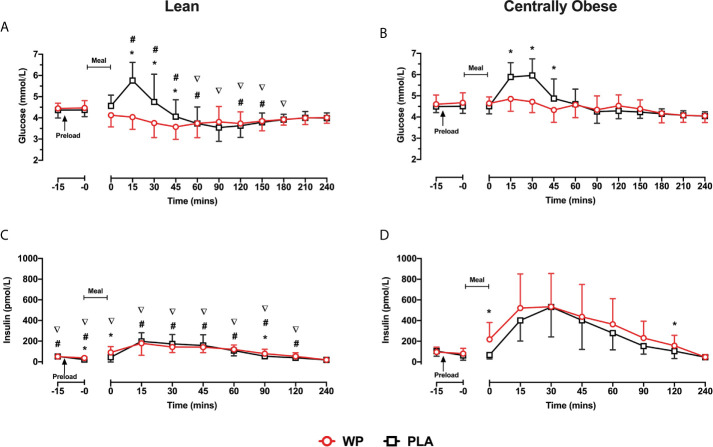
Mean ± SD time-course changes in plasma glucose **(A**, **B)** and insulin **(C**, **D)**, following pre-meal consumption of a WP (**red**) and PLA preload (**black**) in lean **(A**, **C)** and centrally obese **(B**, **D)** males. Pre-meal treatments were administered 10 min before breakfast (t = −10 min), as indicated by the arrow on the figure. The mixed-nutrient meal was served at t = −0min and was to be consumed within 15 min. Time-course glucose data were analysed by a mixed-model ANOVA with repeated measures (time and treatment). Time-course insulin data were analysed by a Friedman’s ranks test with pairwise comparison. Between-group differences in postprandial insulin were assessed by a Kruskal–Wallis H test. *Denotes a within-group treatment effect (i.e., WP vs PLA). ^#^denotes between-group differences (i.e., lean vs centrally obese) during the WP trial. ∇denotes between-group differences during the PLA trial. Statistical significance was accepted as p <0.05.

Fasting plasma glucose concentrations were similar on both study days and did not differ between lean and centrally obese participants. PPG displayed a significant *time* ∗ *treatment* interaction (p <0.0001), such that PPG concentrations were reduced after WP between t = 15–45 min (all p <0.05; **Figures 1A, B**). A significant *time* ∗ *condition* interaction (p = 0.020) was also observed for PPG responses, where obese individuals displayed greater PPG concentrations throughout the postprandial periods compared to lean subjects (all p <0.05; see **Figure 1A** for specific time points). A main effect of *treatment* for PPG AUC_0–60_ was found (p <0.0001), whereby PPG AUC_0–60_ were 18.2 and 13% lower following the WP shot compared to PLA in the lean and centrally obese cohort, respectively (both p = 0.001; **Table 2**). Compared to lean subjects, AUC_0–60_ were ~17% greater in centrally obese subjects (*condition*, p = 0.002; **Table 2**). PPG AUC_0–240_ were unaffected by the WP preload.

**Table 2:** Postprandial area under the curve values of biochemical parameters during the mixed-meal tolerance test.

	Lean (n = 12)		Centrally Obese (n = 12)		P-value		
	WP	PLA	WP	PLA	Treatment	Condition	Treatment*Condition
	**Glucose AUC (mmol/L/min^−1^)****^a^**						
**0–60 min**	3.8 ± 0.5	4.7 ± 0.7*	4.6 ± 0.4^#^	5.3 ± 0.6*^#^	**P <0.0001**	**P = 0.002**	P = 0.468
**0–240 min**	3.9 ± 0.4	4.0 ± 0.4	4.4 ± 0.3^#^	4.5 ± 0.3^#^	P = 0.055	**P <0.0001**	P = 0.715
	**Insulin iAUC (pmol/L/min^−1^)****^a^**						
**0–60 min**	97.0 ± 59.4	102.4 ± 59.9	361.8 ± 226.4^#^	311.1 ± 215.5^#^	P = 0.440	**P <0.0001**	P = 0.353
**0–240 min**	35.1 ± 20.0	30.81 ± 18.1	139.5 ± 90.2^#^	83.3 ± 54.6*^#^	**P = 0.047**	**P <0.0001**	P = 0.379
	**GLP-1 iAUC (pmol/L/min^−1^)****^a^**						
**0–60 min**	23.6 ± 7.4	7.3 ± 5.0*	19.4 ± 7.9	6.7 ± 4.0*	**P <0.0001**	P = 0.217	P = 0.181
**0–240 min**	15.0 ± 4.3	4.7 ± 3.7*	11.8 ± 4.6^#^	5.2 ± 4.1*	**P = 0.001**	**P = 0.040**	**P = 0.011**
	**GLP-1_ACTIVE_ iAUC (pmol/L/min^−1^)****^a^**						
**0–60 min**	3.8 ± 1.9	1.5 ± 1.1*	2.3 ± 1.4	1.1 ± 0.6*	**P <0.0001**	P = 0.196	P = 0.217
**0–240 min**	2.0 ± 0.7	0.7 ± 0.4*	1.4 ± 0.7	0.8 ± 0.5*	**P <0.0001**	P=0.426	**P = 0.043**
	**GLP-1_ACTIVE/TOTAL_ iAUC (pmol/L × min^−1^)****^b^**						
**0–60 min**	4.7 ± 2.6	2.5 ± 1.7*	2.4 ± 1.7^#^	1.9 ± 1.4	**P = 0.009**	**P=0.021**	P = 0.099
**0–240 min**	10.2 ± 6.4	5.2 ± 3.6*	7.1 ± 3.8	5.4 ± 4.7	**P = 0.004**	P = 0.386	P = 0.132
	**GIP iAUC (pmol/L/min^−1^)****^a^**						
**0–60 min**	50.5 ± 19.0	51.8 ± 16.2	57.9 ± 23.0	53.9 ± 23.2	P = 0.650	P = 0.549	P = 0.383
**0–240 min**	36.5 ± 14.8	31.2 ± 13.7	43.3 ± 14.9	33.8 ± 13.7*	**P <0.0001**	P = 0.382	P = 0.247
	**Acetaminophen AUC (mol/L/min^-1^)****^a^**						
**0-60min**	65.1 ± 32.5	100.7 ± 31.1*	58.1 ± 19.7	90.0 ± 20.3*	**P <0.0001**	P = 0.307	P = 0.787
**0-240min**	68.8 ± 14.9	77.5 ± 17.5	60.3 ± 13.1	67.2 ± 11.4	**P = 0.029**	P = 0.067	P = 0.793

Data presented as means ± SD.

a Variables are presented as time-averaged during the early (0 -60 min) and total (0–240 min) postprandial period.

b The iAUC for GLP-1_ACTIVE/TOTAL_ is presented as the as the sum of iAUC over time (i.e., 60 or 240 min) and is thus, not time-averaged.

All data were analysed by a two-way mixed ANOVA. Between and within group differences were analysed if there were any significant main or interaction effects reported from the ANOVA.

*Denotes a within-group treatment effect (WP vs PLA).

^#^Denotes a between-group effect (lean vs centrally obese). Bold values indicate statistical significance from the two-way mixed ANOVA.

#### Plasma Insulin Concentrations

Due to the distribution of data, temporal insulin responses were analysed non-parametrically. During both study days, insulin concentrations increased following breakfast, peaking at t = 15–30 min before returning to baseline values (**Figures 1C, D**). In obese subjects, insulin concentrations were greater at t = 0 min and at t = 120 min following the WP preload compared to PLA. Similarly, the WP shot increased the early (t = −0 min and t = 0 min) and late (t = 90 min) secretion of insulin in lean subjects compared to PLA (all p <0.016, **Figures 1C, D**). Overall insulin secretion (iAUC_0–240_) was greater following the WP shot comparative to PLA (*treatment*, p = 0.047), although upon sub-group analysis, this was only evident in the obese cohort (p = 0.044) and not lean subjects (p = 0.393). As expected with the population studied, centrally obese individuals had greater insulin concentrations during both trials (p <0.0001). Accordingly, insulin iAUC_0–240_ were a ~two-threefold greater in obese participants compared to lean during both trials (p <0.0001; **Table 2**).

#### Plasma Total and Active GLP-1 Concentrations

**Figure 2:**
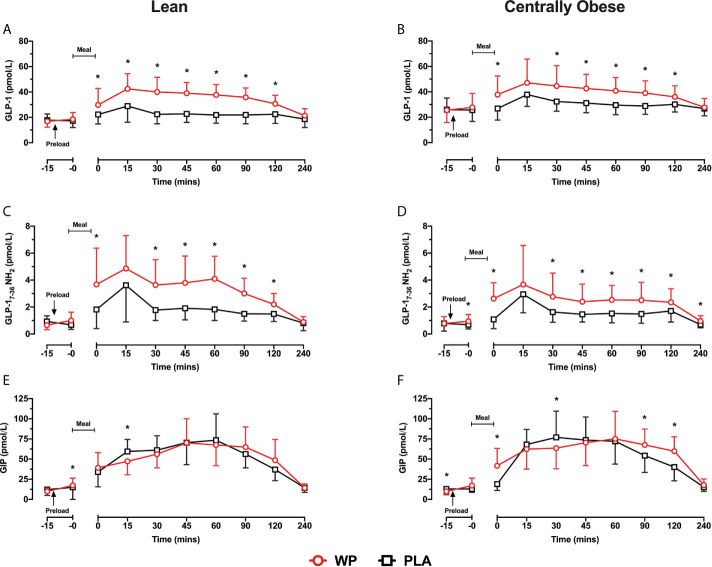
Mean ± SD time-course changes in plasma GLP-1 **(A**, **B)**, GLP-1_ACTIVE_ **(C**, **D)** and GIP **(E**, **F)** following pre-meal consumption of a WP (**red**) and PLA preload (**black**) in lean **(A, C**, **E)** and centrally obese **(B, D, F)** males. Pre-meal treatments were administered 10 min before breakfast (t = −10 min), as indicated by the arrow on the figure. The mixed-nutrient meal was served at t = −0 min and was to be consumed within 15 min. GLP-1 and GIP data were analysed by a mixed-model ANOVA with repeated measures (time and treatment). Time-course GLP-1_ACTIVE_ data were analysed by a Friedman’s ranks test with pairwise comparison to locate within-group treatment effects, and by a Kruskal–Wallis H test to examine between-group differences. *Denotes a within-group treatment effect (p <0.05).

GLP-1 concentrations increased following breakfast consumption on both study days, peaking at t = 15 min before returning to baseline values (**Figures 2A, B**). Compared to PLA, pre-meal WP increased postprandial GLP-1 concentrations (*time* ∗ *treatment*, p <0.0001) with significant differences between t = 0–120 min in both groups (all p <0.034). Postprandial GLP-1 responses were similar between cohorts (*time* ∗ *condition*, p = 0.577), although overall GLP-1 concentrations were ~25% greater in obese individuals (*condition*, p = 0.035). When assessing GLP-1 secretion (i.e., iAUC_0–240_), a significant main effect for *treatment* was found (p = 0.002), such that WP increased GLP-1 iAUC_0–240_ by a two–three-fold compared to the PLA trial in both lean and obese cohorts (p = 0.002 and p = 0.015, respectively). However, GLP-1 secretion in response to the preloads differed between groups (*treatment* ∗ *condition*, p = 0.011). During the WP trial, GLP-1 iAUC_0–240_ was ~27% lower in centrally obese participants compared to the lean (p = 0.001); whereas GLP-1 iAUC_0–240_ were similar between groups during PLA (p = 0.760; **Table 2**).

As expected from the increase in GLP-1, postprandial GLP-1_ACTIVE_ concentrations were elevated during the WP trial compared to PLA (p <0.0001) with significant differences at t = 0 min and between t = 30–120 min in both groups (all p <0.034; **Figures 2C, D**). Accordingly, GLP-1_ACTIVE_ iAUC_0-240_ was increased by a two-threefold during the WP trial compared to PLA (*treatment*, p <0.0001; **Table 2**). A significant *treatment* ∗ *condition* interaction was also found (p = 0.043), which revealed that during the WP trial there was a tendency for GLP-1_ACTIVE_ iAUC_0–240_ to be lower in obese subjects compared to lean (p = 0.085), with no differences during PLA (p = 0.671).

#### Plasma GLP-1_ACTIVE/TOTAL_ Concentrations

Relative to the total amount of GLP-1 secreted during the early postprandial period (0–60 min), the GLP-1_ACTIVE/TOTAL_ iAUC_0–60_ was greater during the WP trial compared to PLA (*treatment*, p = 0.009) with a tendency for similar responses between cohorts (*condition* ∗ *treatment*, p = 0.099). However, when GLP-1_ACTIVE/TOTAL_ iAUC_0–60_ responses were assessed individually by pairwise comparisons, such an effect was only evident in the lean cohort (p = 0.004) and not in obese subjects (p = 0.431). Additionally, it was revealed that the GLP-1_ACTIVE/TOTAL_ iAUC_0–60_ during the WP trial was lower in obese individuals compared to lean participants (p = 0.016), whereas GLP-1_ACTIVE/TOTAL_ iAUC_0–60_ were similar between cohorts during PLA (p = 0.329).

#### Plasma GIP Concentrations

Fasting GIP concentrations were ~23% lower at commencement of the WP trial vs PLA in the centrally obese group (p = 0.040), whereas basal GIP concentrations were similar between trials in lean subjects (**Figures 2E, F**). During both study days, GIP concentrations increased following the breakfast meal before returning to baseline values at t = 240 min. Postprandial GIP concentrations displayed a significant *time* ∗ *treatment* interaction (p <0.0001). In obese subjects, GIP concentrations were elevated at t = 0 min, t = 90 min and t = 120 min during the WP trial compared to PLA (all p <0.001; **Figure 2F**). Similar temporal responses were observed in the lean cohort but without statistical significance (**Figure 2E**). GIP iAUC_0–240_ demonstrated a significant *treatment* effect (p <0.0001), such that GIP iAUC_0–240_ was greater during WP compared to PLA. Upon sub-group analysis, GIP iAUC_0–240_ were increased by 17.7% in the lean group and by 34.3% in the centrally obese group following WP compared to PLA (p = 0.078 and p = 0.001, respectively). GIP iAUC_0-240_ were similar between cohorts during both study days (*condition*, p = 0.382; **Table 2**).

#### Gastric Emptying

**Figure 3:**
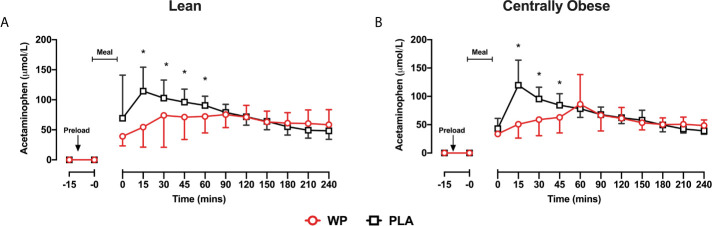
Mean ± SD time-course changes in serum acetaminophen concentrations following the WP (red) and PLA preload (black) in lean **(A)** and centrally obese **(B)** males. Data were analysed by a mixed-model ANOVA with repeated measures (time and treatment). *Denotes a significant within-group treatment effect (p <0.05).

Following breakfast, acetaminophen concentrations increased and remained elevated throughout the trial (**Figure 3**). Serum acetaminophen concentrations demonstrated a significant *time* ∗ *treatment* interaction (p <0.0001), such that acetaminophen concentrations were lower between t = 0–45 min following WP compared to PLA (p <0.0001). As such, acetaminophen AUC_0–60_ were ~35% lower during WP compared to PLA (*treatment*, p <0.0001; **Table 2**). During both study days, gastric emptying rates were similar between subjects. Using pooled data from all participants on both study days, a positive relationship between acetaminophen AUC_0–60_ and glucose AUC_0–60_ was revealed (*r* = 0.318; p = 0.028).

#### Subjective Appetite

There were no differences in individual appetite components following both WP and PLA treatments, thus only combined-appetite scores were analysed. Combined-appetite scores decreased following breakfast in both study days before returning to baseline levels upon trial completion (*time* effect, p <0.001). Combined-appetite scores were unaffected by the WP treatment and displayed similar responses between lean and obese participants (data not shown).

### Metabolic Predictors for GLP-1 Secretion

Since there were differing GLP-1 responses following the WP shot, we assessed potential metabolic predictors for GLP-1 secretion. While there were no correlates found for GLP-1 secretion during PLA (p >0.110), GLP-1 iAUC_0–240_ during the WP trial was negatively correlated with waist-to-hip ratio (r_s_ = −0.431; p = 0.035) and fasting triglycerides (r_s_ = −0.441; p = 0.031). Individuals with a greater HOMA-IR also had lower GLP-1 iAUC_0–240_ following mealtime WP supplementation (r_s_ = −0.419; p = 0.042), but not during PLA. There were no predictors for GIP secretion.

### Preload Palatability and Gastrointestinal Responses

Participants responded positively to the WP preload in terms of taste (76.6 ± 12.4 mm), likability (73.7 ± 11.8 mm) and palatability (77.6 ± 12.7 mm). There were no reported upper or lower gastrointestinal side effects following WP consumption.

## Discussion

We examined the postprandial hormonal and metabolic responses to a mixed-nutrient meal following consumption of novel, ready-to-drink shot containing a low dose of WP (15 g) in lean and centrally obese NGT males. Our primary finding was that consumption of a small pre-meal WP beverage markedly attenuates the rise in PPG in both lean and obese cohorts. These reductions in PPG were associated with the early and sustained secretion of GLP-1, and a slowing of gastric emptying. However, despite comparable reductions in PPG, the metabolism and secretion of GLP-1 following mealtime WP supplementation differs between centrally obese and lean subjects, which may derive from several metabolic complications associated with the viscerally obese state. These data may have important implications for nutritional strategies targeting the biological actions of GLP-1 in abdominally obese and insulin resistant patient populations.

The dose of WP provided, although ~25–65% smaller than those used previously in non-patient populations (24, 38, 39), sufficiently reduced PPG AUC_0–60_ (~13–18%) in both lean and centrally obese adult males. Despite the marked reduction in PPG, pre-meal WP supplementation had minimal effects on insulin secretion across the early postprandial period, which contrasts previous literature (19, 22, 23, 38), suggesting that the observed reductions in PPG were largely insulin-independent. Indeed, gastric emptying, which is a major determinant of PPG (18, 40), was substantially delayed following the WP shot; the latter would also favour the modest insulin responses found (41). Of note, insulin concentrations were elevated immediately post-breakfast (t = 0 min) during the WP trial, which may be in response to an increase in plasma glucagon that can occur following protein feeding (22, 23), although the effects of insulin and glucagon on PPG appear counterbalanced (14). Importantly, our WP shot was just as effective at reducing PPG excursions in centrally obese individuals as it was for lean subjects, which may have significance since our obese cohort exhibited several features consistent with the Metabolic Syndrome (32). For instance, for the same modest elevation in PPG (~8 mmol/L), the incidence of T2D is increased by two-fold in centrally obese individuals with features of the Metabolic Syndrome compared to obese people with more favourable metabolic characteristics (11). Fat accretion around central areas is also associated with increased glycaemic variability (25), which may induce oxidative stress and vascular injury (42), facilitating the onset of CVD. Our data, therefore, supports the application of a low dose of pre-meal WP to regulate PPG in this clinically vulnerable cohort.

Remarkably, PPG concentrations remained largely unchanged from basal values when the mixed-nutrient meal was preceded by the pre-meal WP shot. The magnitude of this suppression was surprising, which may be due to the NGT populations studied, although mealtime WP supplementation (~20 g) has previously failed to produce similar results in lean (38) and obese (24) NGT adults. Additionally, despite upholding NGT, our centrally obese cohort were significantly insulin resistant and may have shown slight glucose intolerance as indicated by the greater post-meal glucose excursions and accompanying hyperinsulinemia compared to lean subjects (43). It is, therefore, unlikely that these findings were due to glycaemic status of our subjects. Of note, an acute dose of exenatide markedly supressed the glycaemic responses to an oral glucose load in centrally obese adults (44), akin to our reported findings. The authors found that exenatide substantially supressed endogenous glucose production [EGP], increased hepatic glucose uptake, and delayed the absorbance of oral glucose (44). Similar mechanisms were also reported in lean adults following sequential glucose loading (45). It is appealing to speculate that our WP preload may have augmented similar gluco-regulatory pathways to those described. In this regard, the increase in GLP-1 following the WP shot may be of particular importance since GLP-1 can inhibit EGP and promote hepatic glucose uptake, independent of its actions on pancreatic islet hormones (46, 47). However, the influence of mealtime WP on hepatic glucose metabolism remains to be characterised and requires future investigation.

The consumption of the pre-meal WP shot differentially affected the secretion of the incretin peptides compared to PLA, such that the WP preload elicited a marked increase in the release of GLP-1 (~127–218% iAUC_0–240_), whereas postprandial GIP responses were modestly affected (~17–34% iAUC_0–240_). This data suggests that GLP-1 plays a greater role in PPG regulation following a low dose of pre-meal WP in NGT adults. Indeed, this assertion is consistent with the observed slowing of gastric emptying during the WP trial, which was most likely mediated by GLP-1 (18, 48). In fact, GLP-1’s influence on gastric emptying may outweigh its insulinotropic effects to regulate PPG (49), where the glucose-lowering potency of GLP-1 is attenuated when its actions to delay gastric emptying are overridden (50). On the other hand, GIP reduces PPG by augmenting insulin secretion in a strict glucose-dependent manner (51). Thus, due to the low PPG concentrations experienced during the WP trial, the “requirement” for the insulinotropic actions of GIP was negligible, which may provide an explanation for the modest postprandial GIP responses observed (41).

In the present study, we confirm previous reports that GLP-1 secretion is disordered in obese individuals (27, 28), particularly in those with abdominal adiposity (52). During the WP trial, GLP-1 iAUC_0–240_ was ~27% lower in centrally obese participants compared to their lean counterparts. However, these findings were surprising given that there were no group differences in GLP-1 secretion during PLA. Gastric emptying rates, which are known to determine GLP-1 release (53), were also similar between groups; although our chosen method to quantify gastric emptying may have been inadequate to distinguish inter-variable differences. Nonetheless, there is no clear evidence to suggest that gastrointestinal motility is disordered or unresponsive to dietary protein-mediated stimulation in obese individuals (54, 55). Therefore, it is unlikely that the reduced GLP-1 secretion observed during the WP trial was due to delayed gastric emptying in centrally obese subjects.

Obesity is associated with the reduced expression of specific L-cell genes, including those associated with nutrient sensing (56), which may provide an explanation for the diminished GLP-1 secretory responses. Explorative analysis revealed that during the WP trial, GLP-1 secretion was inversely related with an individual’s fasting triglyceride concentrations (r_s_ = −0.441) and waist-to-hip ratio (r_s_ = −0.431), whereas no correlates were found during PLA. Insulin resistance was also associated with attenuated GLP-1 responses following the WP shot (r_s_ = −0.419). Given GLP-1 secretion in response to a large dose of hydrolysed WP (~48 g) appears to be intact in obese individuals (55), central adiposity, or its associated metabolic derangements, may compromise the responsiveness of protein-sensing receptors to mediate GLP-1 secretion in response to smaller amounts of dietary proteins/peptones (56, 57). It must be acknowledged that this assertion, although of interest, does not establish a causal relationship and requires future study.

In the current investigation, we measured both the total and active GLP-1 peptide, providing conclusions on the metabolism of GLP-1 following its release (34). As expected, GLP-1_ACTIVE_ concentrations were greater following the WP treatment, which reflects the increase in the total peptide secreted. However, the WP shot also increased the GLP-1_ACTIVE/TOTAL_ ratio suggesting an inhibitory effect on DPP-IV activity, although this was only evident in the lean cohort. Thus, our WP dose (15 g) may have been insufficient to inhibit DPP-IV activity in centrally obese individuals, which is unsurprising given that DPP-IV activity is elevated in obese states (58, 59). This hypothesis is also coherent with previous findings in overweight people with well controlled T2D, where an increase in GLP-1_ACTIVE/TOTAL_ was observed after consumption of a WP preload at a dose three-fold greater than what was administered here (15 g vs 50 g) (21). Nonetheless, the physiological importance of this observation is unclear, particularly given WP reduced PPG in both cohorts, implying that the mechanisms by which mealtime WP regulates glycaemia are at least partially intact in abdominally obese individuals. The regulation of PPG by endogenous GLP-1 is also complex that involves the activation of vagal afferent fibres in the gut, prior to its release and degradation (34), that are not reflected by our venous measures. To challenge this concept and to delineate the therapeutic role WP-mediated GLP-1 secretion on PPG metabolism, it would be worthwhile to examine the application of mealtime WP with and without the concomitant administration of a DPP-IV inhibitor and the GLP-1 antagonist, exendin (9–39) NH_2_.

In both cohorts, subjective appetite parameters were unaffected following the WP preload, which is at variance with some (19) but not all (24, 38) previous studies. These results might be construed as surprising given the increase in GLP-1 observed during the WP trial, where the consumption of WP also stimulates the secretion of several other anorexigenic hormones and supresses postprandial ghrelin concentrations (60, 61). However, the physiological and hormonal changes that result in objective reductions in energy intake are not always noticeable at the subjective level (62). It is, therefore, difficult to conjecture from the subjective markers reported the potential effect of our pre-meal WP shot on subsequent energy intake.

There are few studies that have assessed the long-term application of mealtime WP to regulate glycaemia, which may be in part due to the laborious and inconvenience associated with preparing traditional WP supplements, particularly if these are to be consumed multiple times per day (14). Novel to this study, we used a WP preload created specifically for free-living glycaemic management. Our WP shot contained 15 g of dietary protein from 100 ml of low-viscosity liquid that was importantly both effective in its actions but also highly palatable and convenient in its delivery. The WP preload was presented in contemporary packaging and was served “ready-to-drink”, which has been previously shown to attenuate patient self-consciousness when consuming protein supplements publicly (30). Additionally, the WP shot had a ~6-month shelf-life that, without compromising peptide stability and functionality, was stable at both chilled and at room temperatures. This allows for unrestricted access without the need for immediate refrigeration and may help facilitate mealtime WP’s application in a real-world setting. Further strengthening the ecological validity of our findings, care was taken to provide a commonly consumed breakfast meal, and administering the WP shot as a 10 min preload was a timing that we considered to embody free-living eating patterns.

There are, however, several limitations to this study that merit comment. Firstly, the acetaminophen absorbance test was used to measure gastric emptying, which cannot discriminate between the emptying of liquids or solids and fails to distinguish between the effects of gastric emptying or those from small intestinal glucose absorbance. Nonetheless, our finding agrees with previous interventions that used the gold standard, scintigraphy, to measure gastric emptying following mealtime WP supplementation (20). Secondly, since our assessments were carried out at a single breakfast meal, our reported findings do not represent the glycaemic and hormonal responses that occur at meals consumed later in the day (63). To the best of the authors’ knowledge, no studies to date have examined the effect of circadian rhythm on the application of mealtime WP, which requires future investigation. Finally, we did not assess the application of pre-meal WP on hepatic glucose metabolism or trace PPG fluxes. This would have provided detailed insight into the influence of our small WP preload on PPG metabolism, particularly since the ingestion of WP stimulates glucagon secretion (22) that may counterintuitively affect EGP in insulin-resistant individuals.

In summary, consuming 15 g of dietary protein from a small, contemporary WP shot diminishes PPG excursions in lean and centrally obese males. Providing a WP beverage in a small, ready-to-drink format that encompasses both consumer convenience and preference may be a suitable way to apply this strategy free-living. However, the metabolism and secretion of GLP-1 following mealtime WP supplementation is compromised in centrally obese patients, which requires consideration when applying this novel strategy in abdominally obese individuals. Whether such defects are associated with an individual’s insulin resistance, their obese state or other visceral adiposity-related ailments is unknown.

## Data Availability Statement

The raw data supporting the conclusions of this article will be made available by the authors, without undue reservation.

## Ethics Statement

The studies involving human participants were reviewed and approved by the Faculty of Medical Sciences, Newcastle University, UK. The patients/participants provided their written informed consent to participate in this study.

## Author Contributions

KS, ES, and DW designed the research. KS conducted the research. GT and DA assisted with the research. KS and DW analysed the data. KS wrote the paper. KD, ES, and DW contributed to writing the paper. All authors contributed to the article and approved the submitted version.

## Funding

This work was supported by a grant from Arla Foods Ingredients Group P/S awarded to DW and ES. Arla Foods Ingredients Group P/S. designed the whey protein beverage. Arla Foods Ingredients Group P/S had no role in the collection, analysis, or interpretation of these data. Grant number BH172513.

## Conflict of Interest

Author LHB was employed by company Arla Foods Ingredients Group P/S (Viby J, Denmark).

The remaining authors declare that the research was conducted in the absence of any commercial or financial relationships that could be construed as a potential conflict of interest.

## Abbreviations

AUC, area under the curve; CVD, cardiovascular disease; DPP-IV, dipeptidyl peptidase IV; GIP, Glucose-dependent insulinotropic polypeptide; GLP-1, Glucagon-like peptide 1; iAUC, incremental area under the curve; NGT, normal glucose tolerance; PPG, postprandial glycaemia; T2D, type 2 diabetes; WP, whey protein.
