# Supplementary material for: The Postprandial Glycaemic and Hormonal Responses Following the Ingestion of a Novel, Ready-to-Drink Shot Containing a Low Dose of Whey Protein in Centrally Obese and Lean Adult Males: A Randomised Controlled Trial
Source: Front Endocrinol (Lausanne). 2021 Jun 18;12:696977. doi: 10.3389/fendo.2021.696977 (PMC8253223; doi:10.3389/fendo.2021.696977)
Supplement: Supplementary file 1 [file DataSheet_1.docx]

**Supplementary data**

A battery of tests assessing the WP shot’s viscosity and pH were conducted in-house by the manufacturer (Arla Foods Ingredients Group P/S), whilst simultaneously also assessing the product’s behaviour when subjected to different environmental conditions. Assessing viscometry and rheometry, the shear rate of drinks was assessed at 50s^-1^ for 90s using a rheometer (Anton Paar GmBH, Austria) and measured in triplicate. The stability of the WP shot was also assessed at baseline, 4-, 8-, 16- and 24-weeks following storage at both room temperature (20$℃$) or chilled (5$℃$) environmental conditions (**Supplementary material 1**). Over a 24-week period, the product showed a mean deviation in viscosity of -1.1 ± 5.21 cP and 2.64 ± 9.33 cP when stored at 5$℃$ and 20$℃$, respectively, with no difference between conditions. Viscosity was ~30cP greater when the drink was kept chilled compared to room temperature. Similarly, the product’s pH was also unaffected by temperature or incubation/storage time (chilled: 6.54±0.11 pH; room temperature: 6.55±0.14 pH).


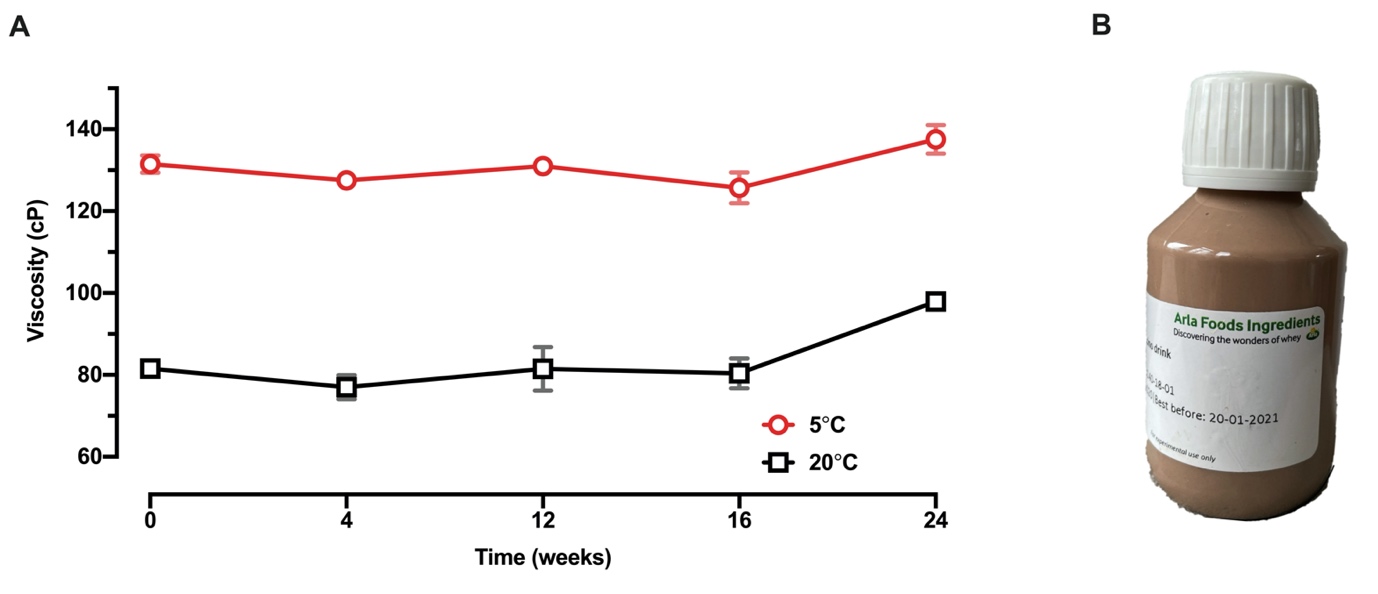


**Supplementary material 1** Mean ± SD time-course changes in viscosity of the Lacprodan® DI-6820 pre-meal shot when kept chilled (5$℃$) and at room temperature (20$℃$) (**A**). Each participant was presented with “cocoa cappuccino” flavoured WP shot in a ready-to-drink format (**B**).

CONSORT flow diagram showing the flow of participants through each stage of a randomised trial.


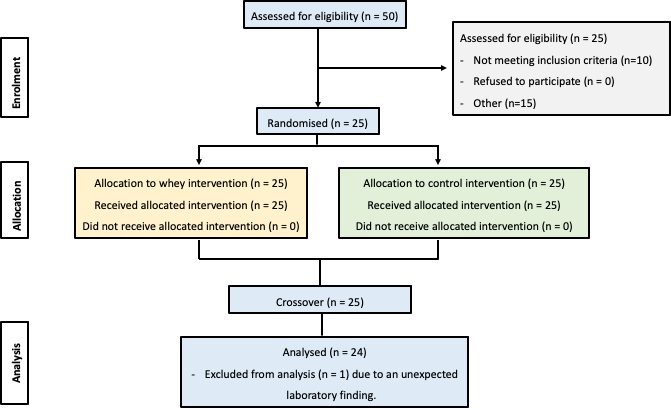


**Supplementary material 2** CONSORT diagram showing the flow of participants through each stage of a randomised trial
